# The effect of Baduanjin on intestinal flora in patients with prediabetes mellitus: Study protocol for a randomized controlled trial

**DOI:** 10.1097/MD.0000000000022108

**Published:** 2020-09-11

**Authors:** Xiangui Lv, Maoyi Yang, Fang Wang, Yao Wang, Xuedong He, Jing Yi, Liping Wang, Shunqi Liao

**Affiliations:** a Chengdu University of Traditional Chinese Medicine; b Hospital of Chengdu University of Traditional Chinese Medicine, Chengdu City, Sichuan, PR China.

**Keywords:** Baduanjin, insulin resistance, intestinal flora, prediabetes

## Abstract

**Background:**

The incidence rate of prediabetes is increasing year by year. Prediabetes is a continuous ever fount of diabetes. Diabetes is closely related to intestinal flora imbalance and insulin resistance (IR). Previous studies have proved that Baduanjin can effectively improve the blood glucose and blood lipid of patients, but there is no relevant research on intestinal flora and IR. Therefore, this study focuses on the influence of Baduanjin on intestinal flora of patients with prediabetes, so as to improve the effect of IR, and finally delay or prevent the occurrence of to diabetes mellitus 2 type (T2DM).

**Methods:**

This study will recruit 80 patients who meet the diagnostic criteria of prediabetes from Hospital of Chengdu University of traditional Chinese Medicine. Eighty patients will be randomly divided into experimental group and control group, 40 cases in each group. The control group received routine lifestyle intervention, and the experimental group received Baduanjin at least 3 to 5 times a week for a total of 6 months. The researchers monitored the intestinal flora, insulin resistance index, blood glucose, blood lipid, body mass index, and other indicators after 3 months of intervention and 6 months of intervention

**Discussion:**

Based on previous studies, intestinal flora is closely related to the occurrence and development of T2DM-IR. Baduanjin can significantly improve the blood glucose and blood lipid of patients with prediabetes, and has a positive effect on the intestinal flora of the elderly and significantly improve the intestinal microecological balance. This study used randomized controlled trial to explore the control method between Baduanjin and conventional lifestyle, in order to further establish the application of Baduanjin in patients with prediabetes.

**Trial registration:**

This trial protocol has been approved by the research hospital and registered in China clinical trial registration center on July 6, 2020 (ChiCTR2000034490).

## 1 Introduction

Prediabetes, defined by blood glucose levels between normal and diabetic levels, is increasing rapidly worldwide. Prediabetes is the subclinical impairment fasting blood glucose (IFG), impaired glucose tolerance (IGT), or both. Prediabetes belongs to the intermediate stage of abnormal glucose metabolism, which is itself a high-risk factor of diabetes. At this time, if proper and regular intervention is given, it can be reversed to normal population.^[1–5]^ Without intervention, 33% of IFG and 65% of IGT population will become diabetes within 6 years.^[6]^ Prediabetes not only directly lead to diabetes, but also increase the risk of retinopathy, peripheral neuropathy, hepatocellular carcinoma, and coronary heart disease.^[7–10]^ Studies have shown that 5% to 10% of people with prediabetes develop diabetes every year.^[11]^ The diabetes conversion rate of IGT alone is 4.35% to 6.35% per year.^[12]^ American ADA expert group pointed out: 70% of patients with prediabetes mellitus (PDM) will eventually turn to diabetes.^[13]^ The Daqing Research Report in China points out that the cumulative incidence of diabetes in 20 years of IGT population is higher than 90%.^[1]^ The International Diabetes Federation (IDF) predicts that the prevalence of IGT will increase globally in the next 20 years, reaching 472 million people by 2030, with the largest absolute increase in Southeast Asia and the Western Pacific. According to the 2012 statistical report, nearly 86 million adult prediabetes patients in the United States.^[14]^ According to the study of South Asia heart metabolism risk reduction center (CARRS), the prevalence of prediabetes in South Asian population was 31.1% to 47.9% in 2011.^[15]^ A cross-sectional survey in 2017 showed that the prevalence of prediabetes in Chinese adults was 35.7%.^[16]^

Prediabetes patients mainly through lifestyle intervention and hypoglycemic drug intervention. Lifestyle intervention mainly includes reasonable diet, proper physical activity, weight control, and so on. Moderate intensity physical activity (such as brisk walking) of 150 minutes/week is beneficial for patients with prediabetes.^[17]^ Moderate intensity physical activity can improve insulin sensitivity and reduce abdominal fat in children and young people.^[18,19]^ Reducing caloric intake is of paramount importance for those at high risk for developing type 2 diabetes (T2DM). Currently, no drug has been approved by the U.S. Food and Drug Administration for the prevention of diabetes. However, the 2018 ADA guidelines point out that metformin can be used for prevention of diabetes in patients with body mass index (BMI) ≥ 35 kg/m^2^, age <60 years old and with a history of gestational diabetes mellitus.^[17]^ There are still some controversies about whether to use drugs in prediabetes. Only those patients who can not adhere to healthy lifestyle for a long time or whose blood sugar still does not drop after lifestyle change should be considered to use hypoglycemic drugs.^[5]^ Lifestyle intervention is the first economic and effective intervention method for patients with prediabetes.

Baduanjin is a moderate intensity aerobic exercise. Baduanjin is based on the theory of Traditional Chinese medicine (TCM) and has a history of more than 1000 years. Baduanjin is very popular in China. Similar to the traditional Taijiquan, Baduanjin has the characteristics of slow movement and relaxation. It is easy to learn and practice and is less likely to cause harm. A set of 8 section brocade qigong practice method, in addition to the starting and stopping movements, also includes 8 movements, each of which is repeated 4 to 8 times. It usually takes about 15 to 20 minutes to complete. Some studies found that through Baduanjin, the blood glucose and blood lipid of PDM patients were significantly improved.^[20]^ Sun Hongmei has proved that Baduanjin has a positive effect on the intestinal flora of the elderly and significantly improves the intestinal microecological balance, which is mainly manifested in the increase of beneficial bacteria Bifidobacterium and Lactobacillus, and the decrease of conditional pathogenic bacteria and harmful bacteria such as Enterobacter, Fusobacterium, and Enterococcus.^[20]^ The fitness effect of Baduanjin was confirmed from the view of microbiology. However, the effect of Baduanjin on intestinal flora of patients with PDM and its correlation with insulin resistance (IR) need to be further studied.

Intestinal flora is a kind of microflora which is located in the human intestinal tract. The intestinal microflora is closely related to the energy metabolism of human body.^[21–23]^ Microbiological studies showed that there were significant differences in the number and species of intestinal flora between diabetes mellitus (DM) patients and normal people.^[24,25]^ Metabolic products produced by microorganisms and host regulation are the key factors affecting IR.^[26]^ The beneficial bacteria in the first gut can correct IR by regulating lipid metabolism of the host. Beneficial bacteria can decompose food components that cannot be digested by the host and convert them into short chain fatty acids. Intestinal probiotics can improve IR by reducing the occurrence of inflammatory reaction.^[27–29]^ In addition, intestinal probiotics can correct impaired glucose tolerance by regulating oxidative stress factors.^[30,31]^ Intestinal flora is closely related to the occurrence and development of T2DM-IR.

We designed a randomized clinical trial to study the effect of Baduanjin on prediabetic patients. The purpose of this study is to determine whether Baduanjin can change the type and quantity of intestinal flora in patients with prediabetes mellitus so as to improve the IR state, and ultimately achieve the recovery of impaired glucose regulation to normal glucose regulation in patients with PDM, effectively delay or even prevent the occurrence of T2DM.

## 2 Methods and analysis

**Figure 1:**
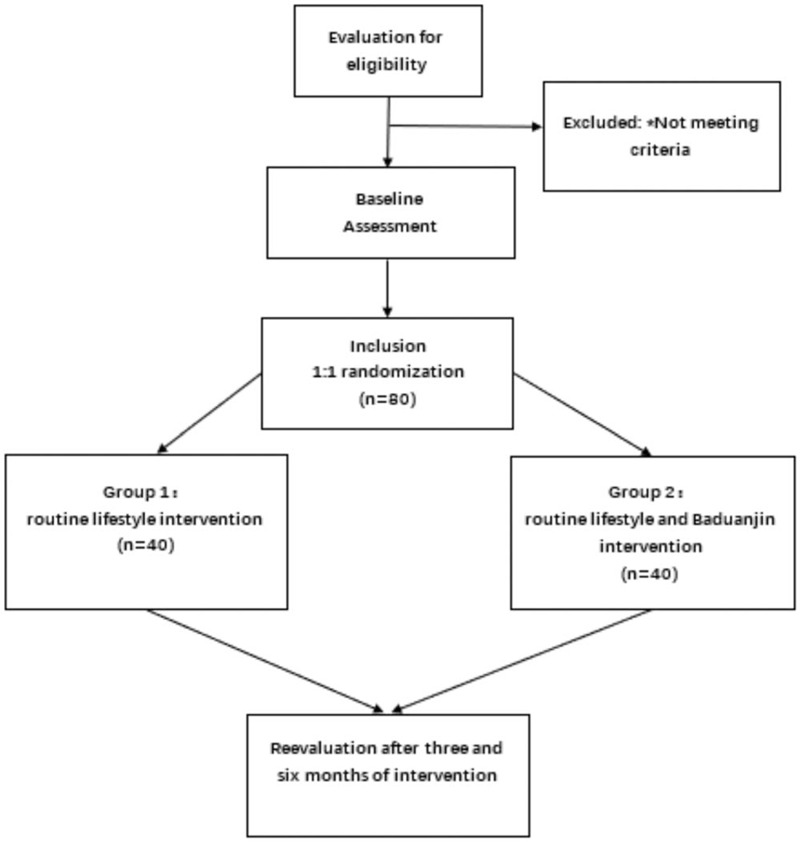
Study flowchart.

The study was registered with the China clinical trials registry (registration number: ChiCTR2000034490). This study is a randomized controlled trial. The research scheme of this research project has been approved by the local ethics committee of Hospital of Chengdu University of traditional Chinese medicine before implementation. It is in line with the provisions of the Helsinki declaration. All subjects will receive written informed consent (Fig. 1).

### 2.1 Site and participant recruitment

The participants will be enrolled from Hospital of Chengdu University of traditional Chinese medicine in Chengdu, Sichuan Province: researchers will conduct trial enrollment publicity through oral publicity, hospital posters, and web ads. Recruitment starts on July 15, 2020 and is expected to be completed on August 15, 2020.

### 2.2 Patient screening

Patients will be deemed eligible after screening according to the inclusion and exclusion criteria.

#### 2.2.1 Inclusion criteria

**Figure 2:**
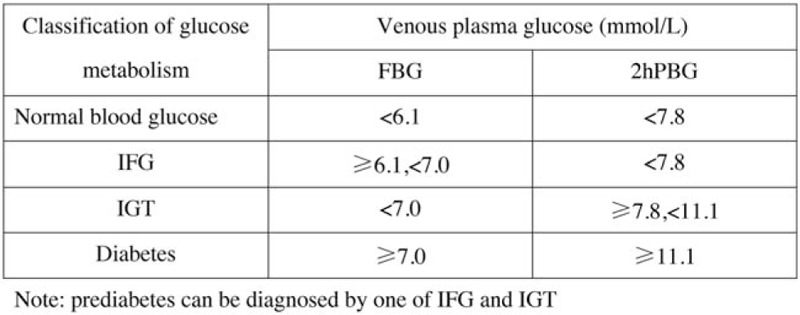
Classification of glucose metabolism status. Prediabetes can be diagnosed by either impaired fasting blood glucose (IFG) or impaired glucose tolerance (IGT).

According to the diagnostic criteria of prediabetes in the 2017 edition of Chinese guidelines for the prevention and treatment of type 2 diabetes mellitus (Fig. 2),^[32]^ all patients underwent oral anhydrous glucose tolerance test (OGTT). The diagnostic criteria of PDM will be as follows:

1. People whose muscle strength is grade IV or above and can complete moderate or above intensity exercise;
2. Aged 18 to 70 years old;
3. They did not keep the habit of regular exercise in recent 3 months (i.e., <3 times a week, each time <30 minutes);
4. Clear consciousness, normal hearing, can communicate with people, understand, and follow the demonstration action;
5. Sign informed consent.

#### 2.2.2 Exclusion criteria

1. Patients who have been diagnosed with DM;
2. Patients with hearing impairment/consciousness impairment/cognitive impairment;
3. All kinds of acute and chronic infection, stress, tumor, and other immune diseases and pregnant or lactating women;
4. Those who need to take the drugs that affect the observation indicators for a long time;
5. The subjects were engaged in severe physical activities;
6. Unwilling to provide personal information.

### 2.3 Randomization and blinding

The patients who passed the qualification assessment will randomly divide the random participants into the Baduanjin group or the routine lifestyle intervention group, and use computer-generated random numbers (FileMarker Pro) for stratification. Data analysts and key outcome assessors will blindly assign treatment assignments. However, some researchers need to conduct the Baduanjin teaching campaign and supervise the experiment, and they will know the group they are assigned to.

### 2.4 Intervention

In addition to the interventions described below, all participants were required to continue their normal life and treatment (i.e., medication) during the trial. All patients participating in the study will participate in Baduanjin for 6 months.

#### 2.4.1 Control group

When the patients are enrolled in the group, they will be given routine lifestyle intervention, including the following aspects:

1. Diet guidance: According to the standard weight (kg = [height (cm) − 105]) and height, activity amount, calculate the daily total calories required by each person. According to nutritionist's recommendation, the distribution of daily total calories: carbohydrate 50% to 60%, protein 15% to 20%, fat 20% to 25%. The total heat of a day can be divided into 1/3 in the morning, 1/5 in the middle, and 2/5 in the evening. The main food is rice, flour, corn, buckwheat, oats, and other coarse cereals; protein should be animal protein combined with plant protein; appropriate eat balsam pear, pumpkin, and other vegetables, choose the fruit with low sugar content. No smoking and alcohol.
2. Exercise guidance: It is suggested that patients choose aerobic exercise as the main exercise mode, such as walking, jogging, and swimming; the exercise intensity should be lower than 100 beats/group; the exercise frequency should be kept at 30 minutes/day, 3 to 5 times/week.
3. Blood glucose monitoring: Teach patients to choose the right blood glucose meter, in the right time according to the correct way of blood glucose monitoring and recording.

#### 2.4.2 Experimental group

**Figure 3:**
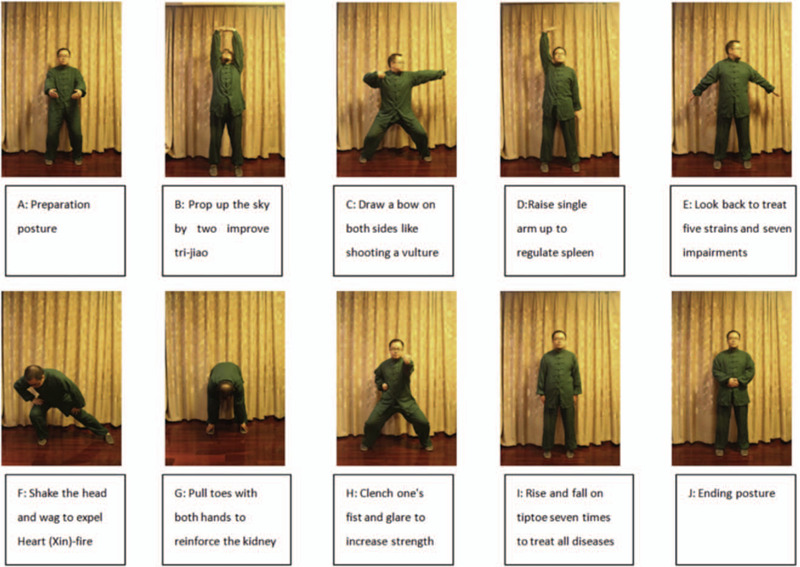
Illustration of the 10 postures of Baduanjin.

Participants in this group will receive routine lifestyle intervention and Baduanjin intervention. Each participant will receive the professional guidance of the coach and require the patients to focus during the exercise. And each participant will master all the movements and practice skilfully. According to the “Fitness Qigong Baduanjin” standard issued by the General Administration of sport of China in 2003, there are 10 movements (Fig. 3). Patients who are unable to complete the whole 8 section brocade Baduanjin project will be excluded from the final analysis.

1. Exercise frequency: At least 3 times a week, 30 to 40 minutes each time;
2. Exercise time: Convenient time for patients;
3. Exercise intervention cycle: 6 months;
4. Sports venues: Practice in groups in nearby Community Health Centre;
5. Sports content: Divided into warm-up part, Baduanjin part, and relaxation part.
  - Warm-up part: Stretch the ligaments of the body to prevent injury during exercise.
  - Baduanjin part: Complete Baduanjin according to the action tips.
  - Relaxation part: Adapt to the change from exercise to stop, eliminate fatigue, and restore physical fitness.
6. Follow-up after exercise: Telephone follow-up once a week, family follow-up once a month, a total of 6 months.

### 2.5 Sample size estimation

Our preliminary study reported that the standard deviation of intestinal microflora in patients with prediabetes was 6.28 CFU/g. At a significant level of 0.05, 80% power was required to detect blood glucose. The average difference between the 2 groups was 5.1 cfu/g. The formula for calculating the sample size is as follows: 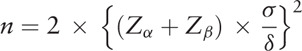


Therefore, we calculated at least 32 sample sizes for each study group, and expected to lose 20% in the future. After calculation, a total of 80 people were admitted.

### 2.6 Outcomes

The indicators of intestinal flora, IR, blood glucose, blood lipid, BMI, anxiety, and sleep status will be collected before, 3, and 6 months after the intervention. The incidence of T2DM will be observed after 1 year follow-up after the intervention.

All indicators will be tested in the Hospital of Chengdu University of traditional Chinese medicine.

#### 2.6.1 Primary outcomes

1. Intestinal flora
  The sterile stool box will be distributed to PDM patients, and the patients were instructed to collect 5 to 10 g stool for the first time in the morning. Sterile feces will be detected by 16S fragment high-throughput sequencing. Through the relevant results of intestinal flora, the number and species of intestinal flora in patients with PDM before and after intervention will be obtained, and the results will be analyzed and compared.
2. IR index
  Fasting venous blood samples will be collected from all participants. Fasting blood glucose (FBG) and fasting insulin (FINS) will be measured by automatic biochemical analyzer (model: AU5800) of Beckman Coulter company. The insulin resistance index (HOMO-IR) will be calculated as an auxiliary measure of insulin sensitivity (HOMO-IR = FBG × FINS/22.5).

#### 2.6.2 Secondary outcomes

1. Fasting blood glucose and 2 hours postprandial blood glucose;
2. The concentrations of triglyceride (TG), total cholesterol (TC), high density lipoprotein cholesterol (HDL-C), low density lipoprotein cholesterol (LDL-C);
3. Changes in body weight (kilogram) and body mass index (kilogram per meter square);
4. Waist to hip ratio;
5. Anxiety was assessed by Hamilton Anxiety Scale (HAMA), that scale includes 14 items;
6. Sleep status assessment: Pittsburgh sleep quality index (PSQI), that scale consists of 18 self-rated items.

### 2.7 Discontinuation and withdrawal

1. Those who are not willing to continue to cooperate with the test and ask to withdraw after joining the group;
2. Poor compliance and failure to complete the intervention as required;
3. The total training times are less than 70% or each training is lower than the required time and intensity.

### 2.8 Data management

Data entry will be conducted by clerks trained in research data entry. All participants will be assigned a participant number, and all data will be stored in a server that only research team members can access. A range check is performed on the data value.

### 2.9 Data analysis

In order to evaluate the effect of Baduanjin intervention and conventional lifestyle intervention, the results will be compared. Spss21.0 will be used for statistical analysis of all the data. The statistical analysis will take bilateral test, and take α = 0.05 as the test level. If the measurement data conform to the normal distribution, the description of 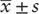 is adopted. If it does not meet the normal distribution, it is described by the median and quartile interval. In the result analysis, if the data conform to the normal distribution, the *t* test of 2 independent samples is used for statistical inference. The Wilcoxon Mann–Whitney rank sum test is used to infer if the distribution does not conform to the normal distribution. The counting data were analyzed by person chi square test.

### 2.10 Patient and public involvement

Patients and/or public were not involved in the design of this study.

### 2.11 Trial monitoring

An independent data monitoring committee (DMC) will be found. The DMC will be responsible to supervise the overall efficacy and safety of the trial. The DMC will have right to decide an interim data analysis and to stop the trial if obvious benefits or harms are observed.

### 2.12 Ethics approval

This trial protocol was approved by the Ethics Committee in the research hospitals: Hospital of Chengdu University of Traditional Chinese Medicine (registration number: 2020KL-011).

## 3 Discussion

The main purpose of this study is to clarify whether Baduanjin can adjust the intestinal flora of patients with PDM, improve the IR state of patients with PDM, regulate the blood glucose and blood lipid of patients with PDM, and finally prevent or delay the occurrence of T2DM.

Based on previous studies, intestinal flora is closely related to the occurrence and development of T2DM-IR. Baduanjin can significantly improve the blood glucose and blood lipid of patients with PDM, and has a positive effect on the intestinal flora of the elderly and significantly improve the intestinal microecological balance.

Therefore, this study will use randomized controlled trial to explore the control method between Baduanjin and conventional lifestyle, in order to further establish the application of Baduanjin in patients with prediabetes. This study will measure changes in intestinal flora, insulin resistance, blood glucose, blood lipids, BMI, waist to hip ratio, anxiety, and sleep status, providing a comprehensive understanding of psychological and physical changes before, during, and after each method and their maintenance. Helping healthcare professionals can choose or offer practices that produce maximum benefit, satisfaction, persistence, and sustainability.

## Author contributions

The protocol was designed by XL and YW under the guidance of FW. All the authors participated in the study. The manuscript was drafted by XL and revised by FW. All authors approved the final manuscript before submission. XL and MY contributed equally to this work and should be regarded as co-first authors.

**Conceptualization:** Yao Wang, Xiangui Lv, Fang Wang.

**Investigation:** Xuedong He, Jing Yi, Liping Wang. Shunqi Liao.

**Project administration:** Fang Wang.

**Supervision:** Fang Wang, Yao Wang.

**Writing – original draft:** Xiangui Lv, Maoyi Yang.

**Writing – review & editing:** Fang Wang, Yao Wang.
